# Massive acute intravascular hemolysis after platelet transfusion: An unrecognized entity

**DOI:** 10.4103/0973-6247.76008

**Published:** 2011-01

**Authors:** Vishakha V. Jain, K. V. Kamesh, S. Sambasivaiah

**Affiliations:** Department of Medicine, Mahatma Gandhi Institute of Medical Sciences, Sewagram, Wardha, Maharashtra -442 102, India; 1 Global Hospital, Lakadi-ka-Pul, Hyderabad, India

Sir,

Transfusion of ABO-mismatched platelets is a common practice when ABO group specific platelet concentrates are unavailable. Acute intravascular hemolysis after platelet transfusion is a rarely encountered entity, but unfortunately a fatal event. We would like that this rare entity of acute hemolytic reactions after platelet transfusions be considered before any platelet transfusions.

A 49-year-old, B Rh positive blood group, female was referred from a district hospital with a history of fever since 5–7 days. There was no history of jaundice, rash, bleeding tendencies, chest pain, dyspnea, or urinary complaints. She was treated at district hospital, was detected to have thrombocytopenia, and was transfused with Platelet rich plasma (PRPs). She received total of 8 PRPs (unmatched) over 2 days. Immediately after the last PRP, patient started complaining of increasing fever with chills, back ache, and started passing red colored urine. Subsequently over the next few hours, she started having jaundice and was referred to our hospital. She had not received any whole blood or packed cells.

At admission to our hospital patient was febrile, jaundiced, no rashes, no evidence of any subcutaneous or submucosal bleed, with unremarkable systemic examination and red colored urine. Her blood which was drawn for investigation showed red coloured sera indicative of gross hemolysis. She was detected to have hemoglobin of 10 g/dl, high WBC counts of 48,470 with thrombocytopenia (platelets 73,000), spherocytes in peripheral smear, hyperglycemia (RBS 284), indirect hyperbilirubinemia, and acute kidney injury (urea 58 and serum creatinine 2.2). Her urine yielded high positive reaction for hemoglobin. Direct antiglobulin test was positive. Her reticulocyte count was 3.5. Serological tests for malaria and dengue were negative.

Subsequently the patient continued to have fever, continued to pass red colored urine, and became tachypnoiec. Her kidney function had deteriorated with blood urea 107 and serum creatinine 5.7. She was immediately started on hemodialysis. Her subsequent reports showed that there was a fall in hemoglobin by 5 g/dl (Hb=5.7 g/dl), hematocrit 16.7, and platelets were 1 lakh. She was transfused 5 units of compatible packed cells. She continued to be tachypnoiec and was destaurating, with ensuing metabolic acidosis. She was intubated and supported by mechanical ventilation. Patient continued to deteriorate and finally died with refractory metabolic acidosis, hyperkalemia, and massive intravascular hemolysis.

The clinical and biochemical scenario presented in this case is consistent with acute hemolytic reaction after PRP transfusions. Transfusion of ABO-mismatched platelets is a common practice when ABO group specific platelet concentrates are unavailable or a histocomapatibility leukocyte antigen (HLA) match is done. Minor ABO incompatibilities between donor’s plasma and recipient’s RBCs are low risk for hemolytic reactions. Blood and blood products from so-called “universal donors” prove catastrophic. These universal donors may have high titers of ABO isohemagglutinins either from vaccinations or other antigen exposures.[1]

There has been evidence of acute hemolytic transfusions due to this ABO incompatibility and some authors have suggested that transfusion of group O components to group A or B patients should only be done if the titer of isohemagglutinins is less than the critical level. The most commonly cited titer is 200 when tested in saline.[2]

In a 5-year review during which time nearly 1000 single-donor ABO incompatible plateletpheresis products were transfused to bone marrow transplant recipients, Shanwell *et al*. noted positive direct antiglobulin tests caused by passively absorbed anti-A and/or anti-B from the donor plasma, but found no cases of hemolysis.[3] In addition, a retrospective review by Mair and Benson showed no significant decrease in hemoglobin, as evidence of hemolysis, with transfusion of ABO-incompatible plateletpheresis products in 24 non-group-O bone marrow transplant patients.[4] At two separate large institutions, the incidence of hemolytic reaction from transfusion of ABO-incompatible single-donor apheresis products was only 0.01% over a 9–10-year period.[3,5]

Nevertheless, many a time platelet transfusions are given to patient from out of their group and most of them are not guided or warranted by evidence-based practice guidelines. We would like that this rare entity of acute hemolytic reactions after platelet transfusions be considered before any platelet transfusions.

Several ways to help minimize the risk may include the following: (1) limiting the quantity of platelet transfusions to what is clinically necessary; (2) continually evaluating those patients receiving large volumes of platelets; (3) whenever possible, transfusing ABO-compatible platelets; and (4) reducing the volume of incompatible plasma transfused for group A, B, or AB patients who must receive ABO-incompatible platelet products, particularly in children and patients with small plasma volume.
